# Accumulation of heme biosynthetic intermediates contributes to the antibacterial action of the metalloid tellurite

**DOI:** 10.1038/ncomms15320

**Published:** 2017-05-11

**Authors:** Eduardo H. Morales, Camilo A. Pinto, Roberto Luraschi, Claudia M. Muñoz-Villagrán, Fabián A. Cornejo, Scott W. Simpkins, Justin Nelson, Felipe A. Arenas, Jeff S. Piotrowski, Chad L. Myers, Hirotada Mori, Claudio C. Vásquez

**Affiliations:** 1 Facultad de Química y Biología, Universidad de Santiago de Chile, Santiago 9170022, Chile; 2 University of Minnesota-Twin Cities, Bioinformatics and Computational Biology, Minneapolis, Minnesota 55455, USA; 3 Yumanity Therapeutics, Cambridge, Massachusetts 02139, USA; 4 University of Minnesota-Twin Cities, Department of Computer Science and Engineering, Minneapolis, Minnesota 55455, USA; 5 Graduate School of Biological Sciences, Nara Institute of Science and Technology, Ikoma 630-0101, Japan

## Abstract

The metalloid tellurite is highly toxic to microorganisms. Several mechanisms of action have been proposed, including thiol depletion and generation of hydrogen peroxide and superoxide, but none of them can fully explain its toxicity. Here we use a combination of directed evolution and chemical and biochemical approaches to demonstrate that tellurite inhibits heme biosynthesis, leading to the accumulation of intermediates of this pathway and hydroxyl radical. Unexpectedly, the development of tellurite resistance is accompanied by increased susceptibility to hydrogen peroxide. Furthermore, we show that the heme precursor 5-aminolevulinic acid, which is used as an antimicrobial agent in photodynamic therapy, potentiates tellurite toxicity. Our results define a mechanism of tellurite toxicity and warrant further research on the potential use of the combination of tellurite and 5-aminolevulinic acid in antimicrobial therapy.

## Abstract (web summary)

The mechanisms of action of the antibacterial metalloid tellurite are unclear. Here, the authors show that tellurite induces an accumulation of hydroxyl radical and intermediates of heme biosynthesis in *E. coli*, and that the heme precursor 5-aminolevulinic acid potentiates tellurite toxicity.

For over 50 years, the oxyanion tellurite (TeO_3_^2−^) has been known as a potent antibacterial^1^,^2^,^3^, ten times more toxic than mercury (Hg^2+^) and several orders of magnitude more than other metals^4^. However, to date, the molecular basis of tellurite toxicity and resistance remains unclear.

Genetic determinants for tellurite resistance have been identified in both plasmids and the bacterial chromosome, including the *ter*, *kilA*, *tehAB*, *telAB* and *ars* operons^5^,^6^,^7^,^8^,^9^,^10^, although the mechanism by which they act is unknown. Studies have suggested that enzymes of central metabolism are required for tellurite resistance^11^. When expressed in *E. coli*, the products of the *ars* and *tehAB* operons do not increase tellurite efflux or alter its uptake^12^, ruling out some potential resistance mechanisms. Recent studies identified several proteins that interact with TerC in *E. coli*^13^, which could improve the understanding on how TerC mediates tellurite resistance.

The current hypothesis of tellurite toxicity in bacteria is based on observations that tellurite depletes thiols^14^, which alters the reduction potential of the cell^15^, and elicits the production of reactive oxygen species (ROS) and ROS-detoxifying enzymes^16^,^17^. This hypothesis proposes that the main causes of tellurite toxicity is its oxidant nature^11^. Proteomic and transcriptomic studies in *E. coli* that identified enzymes required for ROS detoxification among the highest expressed after tellurite exposure support this hypothesis^18^,^19^.

However, despite the evidence suggesting a role for ROS detoxification and thiols in tellurite resistance, some studies have challenged this idea. Inactivating GshA or TrxA in *E. coli* increased tellurite resistance in the presence of selenite^20^, suggesting that higher levels of glutathione and thioredoxin might be detrimental to tellurite resistance. Additionally, there is no definitive evidence linking metal toxicity to ROS production. For example, selenite, which is less toxic than tellurite by several orders of magnitude^4^, elicits higher ROS production than tellurite in *E. coli*^20^. Also, tellurite is still toxic under anaerobic conditions^21^. Further, organisms that are ROS-resistant like *Deinococcus radiodurans* show only modest tellurite resistance^15^.

A major hurdle towards identifying tellurite resistance mechanisms and its toxicity has been the lack of truly resistant organisms for which genetic tools are available. While several studies characterized highly tellurite-resistant organisms, the genetic determinants and mechanisms of tellurite resistance were not identified^22^,^23^. To date, studies addressing this issue have been conducted with organisms that show modest or no tellurite resistance, making it difficult to identify the cellular target(s) and the true resistance mechanisms. To overcome this, in the present work, we developed an unbiased, genome-wide approach to understand tellurite toxicity and resistance. Using directed evolution and chemical genomics independently, we identified the heme biosynthetic pathway as a tellurite target. We confirmed that tellurite elicits the accumulation of protoporphyrin IX, which was correlated with iron depletion and cell death. Tellurite resistance required limiting substrate availability for the last steps of heme biosynthesis, where protoporphyrin IX (from here on proto IX) is synthesized. Increased hydrogen peroxide (H_2_O_2_) or superoxide (O_2_^·−^) detoxification or resistance was not required for tellurite resistance. In fact, tellurite-resistant strains were highly sensitive to H_2_O_2_, indicating that tellurite, H_2_O_2_ and O_2_^·−^ resistance are not directly correlated. Finally, we discovered that micromolar concentrations of the heme biosynthetic precursor 5-aminolevulinic acid (ALA) potentiate tellurite toxicity under both aerobic and anaerobic conditions. Interestingly, ALA is widely used as a prodrug in photodynamic therapy (PDT) for the control of bacteria, parasites, fungi and so on^24^, suggesting that the combined use of tellurite and ALA could further improve the efficacy of PDT and, perhaps, be applied in other biocontrol therapies.

## Results

### Limiting ALA availability increases tellurite resistance

**Figure 1:**
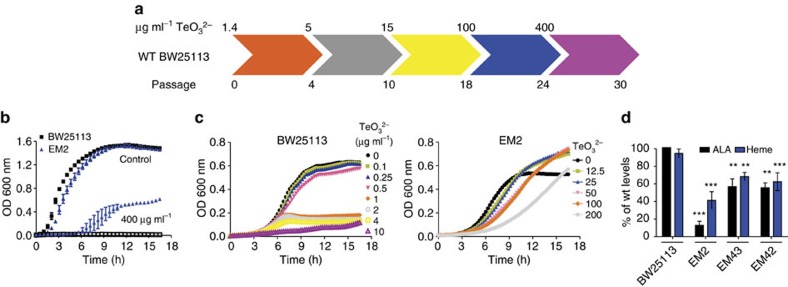
Selection of mutants with increased tellurite resistance. (**a**) Summary of directed evolution experiment in LB medium. The number of serial passages (one passage=growth for 24 h) and the concentration of tellurite used is indicated. Strain EM2 was isolated at passage 26. Representative growth curves of strains BW25113 and EM2 in (**b**) LB or (**c**) M9 medium in the presence of the indicated tellurite concentrations. Values represent the mean of three biological replicates. In (**c**) s.d. was removed for clarity (growth curves with s.d. are shown in Supplementary Fig. 2). (**d**) The indicated strains were grown in M9 medium and the heme and ALA content was measured in cell extracts. The level of heme or ALA in strain EM2, EM42 and EM43 were compared to strain BW25113, which was arbitrarily set to 100%. Values represent the mean of three biological replicates. Error bars represent s.d. TeO_3_^2−^: tellurite **P<0.001; ***P<0.0001 as determined by *t*-test.

To understand the mode of action and cellular targets of tellurite, we performed directed evolution experiments to isolate spontaneous *E. coli*-resistant mutants that showed robust growth in lysogeny broth (LB) medium containing increasing concentrations of tellurite up to 400 μg ml^−1^ (Fig. 1a). We isolated clonal strains from the evolved cultures by plating aliquots on LB plates and selecting isolated colonies from each day for further study. Individual colonies were tested for resistance following growth in LB medium (Fig. 1b and Supplementary Fig. 1a) containing increasing concentrations of tellurite. Three strains with low (15 μg ml^−1^, strain EM40), intermediate (100 μg ml^−1^, strain EM 41) or high resistance (400 μg ml^−1^, strain EM2) were selected for genomic sequencing (Supplementary Table 1) together with the ancestral strain (BW25113).

Sequencing of the evolved strains revealed that mutations accumulated sequentially (Supplementary Table 1) and that they additively increased tellurite resistance (Fig. 1b and Supplementary Fig. 1a). We selected strain EM2 for detailed study because it tolerated high tellurite concentrations in both LB (up to 400 μg ml^−1^; Fig. 1b) and M9 0.2% glucose medium (up to 200 μg ml^−1^; Fig. 1c and Supplementary Fig. 2), levels comparable to other resistant organisms^25^. Unless otherwise indicated, all subsequent experiments were conducted in M9 0.2% glucose medium (from here after referred as M9) to avoid possible chelation of tellurite by components present in LB medium, which could mask the effects of tellurite, as shown to occur with other metals^26^. Mutations found in strain EM2 (Supplementary Table 1) affected genes encoding proteins of pathways including transcription (*rpoD*), response to membrane stress (*pspF*), synthesis of ALA (*hemA* and *hemL*) and the hypothetical protein YigE (Table 1 and Supplementary Table 1). Resistance conferred by the mutations in *rpoD* and *pspF* likely act by inducing transcriptional reprogramming and by ameliorating the effect of tellurite on the *E. coli* cell membrane, respectively^27^,^28^. The role of the hypothetical protein YigE in tellurite resistance remains unclear, and further studies are being conducted to understand its role. The other two mutations conferring tellurite resistance are directly related to heme biosynthesis; *hemA* and *hemL* encode glutamyl tRNA reductase and glutamate semialdehyde 2,1-aminomutase, respectively, which catalyse the synthesis of ALA, the precursor of the pathway.

To determine the individual contributions of the observed mutations on tellurite resistance, we restored each mutation to the wild-type allele in strain EM2. Restoring the *hemA* mutation in strain EM2 with the wild-type allele (strain EM42) strongly reduced tellurite resistance and even impaired growth in the absence of any stress, as reflected by a longer lag phase and decreased growth. Conversely, restoring the wild-type *hemL* allele (strain EM44) in strain EM2 did not decrease tellurite resistance (Supplementary Fig. 1b), suggesting that the activity of the enzyme was not affected, consistent with a conservative amino-acid replacement. Introducing the *hemA*[31E] allele in an isogenic wild-type strain (strain EM43) was not sufficient to restore resistance of strain EM2 (Supplementary Fig. 1b), indicating that the genetic background is important for the observed tellurite resistance. Individually restoring the *pspF* or *rpoD* mutations in strain EM2 (strains EM70 and EM64, respectively) decreased tellurite resistance by twofold as compared with strain EM2 (Supplementary Fig. 1b); however, restoring them together (strain EM71) had an additive effect and reduced growth under all the tellurite concentrations tested (Supplementary Fig. 1b). Together, our results indicate that the *hemA* mutation in strain EM2 shows a dominant effect on the increased tellurite resistance and suggests that altering the levels of ALA and/or heme is required for this process. It can be inferred that the mutation of *hemA* in strain EM2 did not cause a loss of function, as a null mutant requires the supplementation of exogenous ALA for growth and cannot synthesize heme^29^.

To test if the mutation of *hemA* in strain EM2 altered ALA and/or heme levels, these metabolites were quantified in strains BW25113, EM2, EM42 and EM43. The levels of ALA and heme were reduced in strains carrying mutant versions of HemA. In strains EM2 and EM43, the levels of ALA were 13% and 55% of those observed in strain BW25113, and heme was 40% and 68%, respectively (Fig. 1d). Strains carrying wild-type alleles of *pspF*, *rpoD* or *hemL* showed similar levels of heme than strain EM2, confirming that the *hemA* mutation caused the decreased levels of heme (Supplementary Fig. 1c). In summation, our results indicate that the mutation in *hemA* is responsible for the decreased levels of ALA and heme, which in turn increased tellurite resistance.

### Chemical genomic analysis of tellurite toxicity

**Figure 2:**
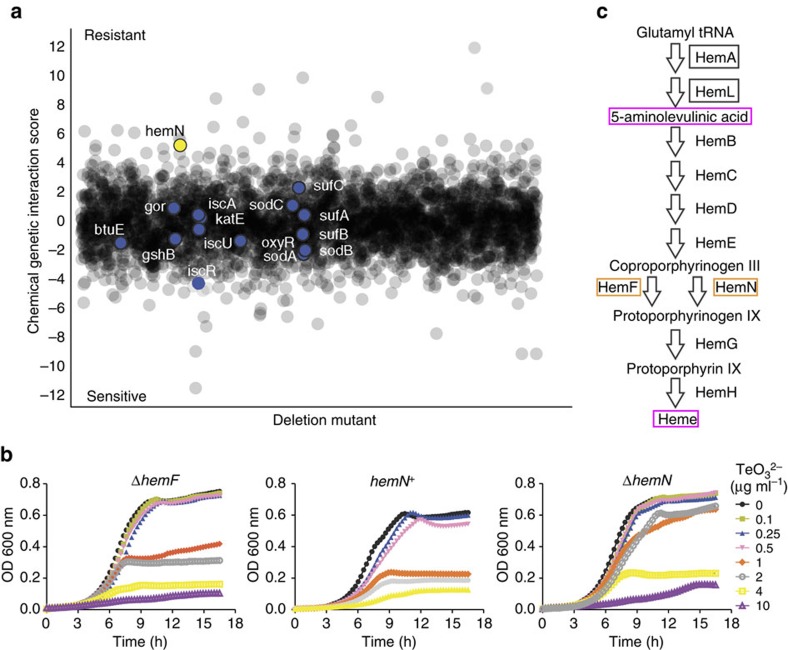
Chemical genomics of tellurite. (**a**) Treatment of the *E. coli* barcoded deletion collection with tellurite. Positive or negative chemical genetic interaction scores represent increased or decreased tellurite resistance, respectively. Selected deletion mutants of genes encoding proteins required for heme biosynthesis (*hemN*; yellow circle), glutathione metabolism (*gor*, *gshB*), ROS resistance (*btuE*, *oxyR*, *sodA*, *sodB sodC*, *katE*) and iron–sulfur cluster assembly (*iscA*, *iscR*, *iscU*, *sufA*, *sufB*, *sufC*) are indicated. (**b**) Representative growth curves of strains Δ*hemF*, *hemN*^*+*^ and Δ*hemN* in M9 medium in the presence of the indicated tellurite concentrations. Values represent the mean of three biological replicates. s.d. was removed for clarity (growth curves with s.d. are shown in Supplementary Fig. 4). (**c**) Schematic representation of heme biosynthetic pathway in *E. coli* BW25113. Selected proteins encoded by genes that when inactivated (orange box) or mutated (black box) increased tellurite resistance or heme biosynthetic intermediates (magenta box) that showed altered levels in strain EM2 are highlighted. TeO_3_^2−^: tellurite.

As a complementary approach to understand tellurite toxicity, we used chemical genomic profiling of *E. coli*^30^ to identify deletions that are responsive to tellurite. We challenged a pooled collection of ∼6,000 deletion mutants with either tellurite or a solvent control (dimethylsulfoxide (DMSO)) in LB medium. Sequencing of strain-specific barcodes allowed us to determine the fitness of each mutant strain in the presence of tellurite relative to the solvent control (Fig. 2a and Supplementary Data 1).

Deletion mutants for 377 and 130 genes showed a significant threefold increase or decrease in fitness after tellurite exposure (Fig. 2a and Supplementary Data 1), respectively. Among the top deletion mutants sensitive to tellurite, we detected significant enrichment (*P*<0.001, hypergeometrical test) for genes involved in the Kyoto Encyclopedia of Genes and Genomes pathway of flagellar assembly (eco02040). Unexpectedly, no deletion mutants annotated to degradation and response to H_2_O_2_ or O_2_^·−^, iron sulfur cluster assembly, or glutathione metabolism, were among the top sensitive strains (Fig. 2a and Supplementary Data 1). Supporting these results, we found that catalase activity was fivefold lower in strain EM2 than in strain BW25113, and did not change with either H_2_O_2_ or tellurite exposure. Further, superoxide dismutase activity, tellurite reductase (TR) activity and thiol levels were similar between both strains (Supplementary Fig. 3a,d,e). Consistent with the lower levels of catalase activity, strain EM2 was more sensitive to H_2_O_2_ than strain BW25113 (Supplementary Fig. 3b,c). ALA supplementation restored catalase activity in strain EM2 to wild-type levels, indicating that the decreased activity was due to lower heme content (Supplementary Fig. 3a). Previously, it was proposed that H_2_O_2_ and O_2_^·−^ are key intermediates in tellurite toxicity, and that decreasing their levels and maintaining the pool of reduced glutathione are required for tellurite resistance^14^,^17^. However, our results provide evidence that there is no direct correlation between thiol levels, H_2_O_2_, O_2_^·−^ and tellurite resistance.

Among the top deletion mutants resistant to tellurite, we detected enrichment for genes involved in the gene ontology category of cofactor biosynthesis (*P*<0.01; gene ontology: 0051188; hypergeometrical test) and in the Kyoto Encyclopedia of Genes and Genomes pathway of porphyrin and chlorophyll metabolism (*P*<0.05; hypergeometrical test). In both cases, a deletion strain for a gene implicated in heme biosynthesis, *hemN*, was included (Fig. 2a and Supplementary Data 1). HemN is an oxygen-independent coproporphyrinogen III (copro III) dehydrogenase that is functional under both aerobic and anaerobic conditions^31^,^32^. It is not surprising that no other deletion strains for genes encoding enzymes of heme synthesis were found in the analysis, as most of these enzymes are essential for aerobic growth in the LB medium^33^. Nevertheless, because the genome of *E. coli* encodes two enzymes able to convert copro III to protoporphyrinogen IX that can be individually inactivated, namely HemN and HemF, it allowed testing if deleting genes downstream of *hemA* also increased tellurite resistance. We confirmed the resistance of the individual Δ*hemF* and Δ*hemN* strains and found that they were able to grow in medium containing up to 2 and 4 μg ml^−1^ tellurite, respectively (Fig. 2b and Supplementary Fig. 4). Complementation of strain Δ*hemN* (strain *hemN*^+^) restored tellurite sensitivity to wild-type levels (Fig. 2b and Supplementary Fig. 4), confirming the observed result.

Taken together, the chemical genomic analysis and directed evolution approach indicate that limiting substrate availability for the last two steps of heme biosynthesis increased tellurite resistance (Fig. 2c). Based on these results, we speculated that tellurite might either interact with metabolites downstream of copro III or cause their accumulation, which could result in cell death.

### Tellurite induces the accumulation of heme intermediates

**Figure 3:**
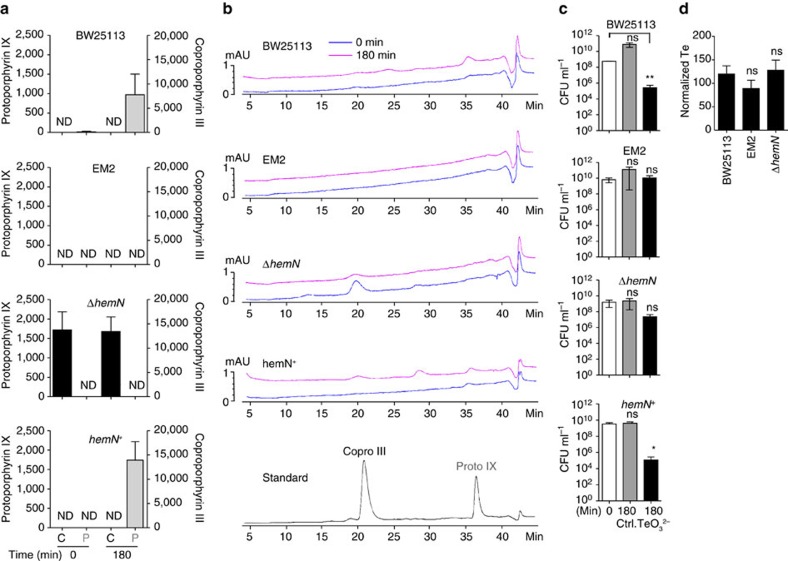
Proto IX accumulates in tellurite exposed cells. (**a**) Strains BW25113, EM2, Δ*hemN* and *hemN*^*+*^ were grown in M9 medium, challenged with tellurite (25 μg ml^−1^) and the porphyrin content of the cell was extracted and quantified by HPLC. In parallel, commercially available standards were quantified and used to identify each peak. Proto IX represents the sum of protoporphyrinogen IX and proto IX. Units for proto IX and copro III are nanomoles normalized by mg of protein. Time under the graph represents the time of porphyrin extraction with respect to tellurite exposure. C=coproporphyrin III (black bars); P= proto IX (gray bars). Values represent the mean of 3 biological replicates. Error bars represent s.d. ND=not detected. (**b**) Representative HPLC chromatograms of samples from each strain quantified in **a**. Blue (0 min) and magenta lines (180 min) in each chromatogram represent the time at which porphyrins were extracted with respect to tellurite addition. (**c**) CFU ml^−1^ were quantified from the same samples used for porphyrin extraction shown in **a**. Ctrl=untreated cells. Time under the graph represents the time at which CFU ml^−1^ were determined with respect to tellurite exposure. Values represent the mean of three biological replicates. Error bars represent s.d. ns, not significant; *P<0.05; **P<0.001 as determined by *t*-test. (**d**) ICP-OES analyses. Strains BW25113, EM2 and Δ*hemN* were grown in M9 medium, challenged with tellurite (25 μg ml^−1^) and intracellular tellurium was quantified by ICP-OES analysis after 180 min. Units for normalized Te are μg ml^−1^ of tellurium g of cell pellet^−1^. Values represent the mean of three biological replicates. Error bars represent s.d. ns: not significant as determined by *t*-test. TeO_3_^2−^: tellurite.

Following the logic that tellurite exposure could lead to the accumulation of heme intermediates downstream of the reaction catalysed by HemN and HemF, we quantified the levels of copro III and proto IX in cells exposed to tellurite. Because protoporphyrinogen IX spontaneously oxidizes to proto IX on extraction^34^, the values presented represent the sum of both intermediates. In BW25113, the levels of proto IX increased 60-fold after tellurite exposure (Fig. 3a,b) and cell viability decreased by 2,000-fold (Fig. 3c). Conversely, proto IX was undetectable in strains Δ*hemN* and EM2, and only copro III was detected in strain Δ*hemN*, although the levels remained unchanged after tellurite exposure (Fig. 3a,b). As expected, tellurite did not affect the growth of strain EM2, and the viability of strain Δ*hemN* only decreased by 70-fold (Fig. 3c), a lower reduction than for strain BW25113. Complementing strain Δ*hemN* resulted in wild-type levels of porphyrins and increased tellurite sensitivity (Fig. 3a–c). No significant changes in the levels of intracellular tellurite were observed between the tested strains (Fig. 3d), ruling out that the differences in the accumulation of heme intermediates and in cell viability were caused by decreased exposure to the toxic compound.

### ALA potentiates tellurite toxicity

**Figure 4:**
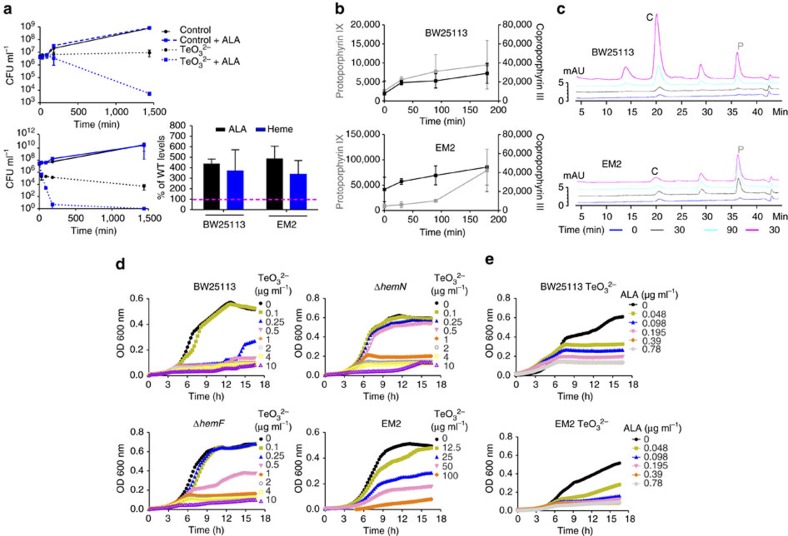
ALA potentiates tellurite toxicity. (**a**) Strains BW25113 (upper panel) and EM2 (lower panel) were grown in M9 medium supplemented or not with 25 μg ml^−1^ ALA, challenged or not with tellurite and CFU ml^−1^ were quantified at the indicated time points. Strains BW25113 and EM2 were exposed to 10 and 400 μg ml^−1^ tellurite, respectively. Heme and ALA content were determined from cell extracts. Levels of heme and ALA in strains BW25113 and EM2 were compared with those of strain BW25113 grown without ALA (dotted magenta line, from Fig. 1d). Values represent the mean of three biological replicates. Error bars represent s.d. (**b**) Strains BW25113 and EM2 were grown in M9 medium supplemented with 25 μg ml^−1^ ALA and challenged with tellurite (25 and 400 μg ml^−1^ for strains BW25113 and EM2, respectively) and the porphyrin content was extracted and quantified by HPLC at the indicated time points. Units for proto IX and coproporphyrin III are nanomoles normalized by mg of protein. Values represent the mean of three biological replicates. Error bars represent s.d. (**c**) Representative HPLC chromatograms of samples from each strain quantified in **b**. The colour of each chromatogram (indicated at the bottom of the figure) represents the time at which porphyrins were extracted regarding to tellurite addition. C=coproporphyrin III; P=protoporphyrin IX. (**d**) Representative growth curves of strains BW25113, EM2, Δ*hemN* and Δ*hemF* in M9 medium supplemented with 25 μg ml^−1^ ALA in the presence of the indicated tellurite concentrations. Values represent the mean of three biological replicates. S.d. was removed for clarity (growth curves with s.d. are shown in Supplementary Fig. 5). (**e**) Representative growth curves of strains BW25113 and EM2 in M9 medium with tellurite (0.5 and 50 μg ml^−1^, respectively) supplemented with the indicated concentrations of ALA. Values represent the mean of three biological replicates. S.d. was removed for clarity. Growth curves with s.d. are shown in Supplementary Fig. 6). TeO_3_^2−^: tellurite.

An alternative approach to test if tellurite toxicity is caused by the accumulation of intermediates in heme biosynthesis is to increase the availability of substrates for the pathway. In *E. coli*, ALA, the precursor of the pathway, is transported by the dipeptide transport system encoded by the *dpp* operon^35^ and can be used for this purpose. The rationale is that if strain EM2 acquired at least part of tellurite resistance by limiting ALA levels, then supplementing the growth medium with exogenous ALA should bypass the effect of the *hemA* mutation and decrease tellurite resistance. To compare directly the effect of ALA on the growth of strain BW25113 and EM2, the concentration of tellurite was increased for strain EM2 to levels (400 μg ml^−1^) that resulted in a similar decrease in cell viability than those observed for strain BW25113 (Fig. 3c). Sublethal concentrations of ALA (25 μg ml^−1^) strongly sensitized strain EM2 to tellurite and cell viability decreased to almost undetectable levels after 180 min of exposure (Fig. 4a). In contrast, EM2 cells grown in the absence of ALA and exposed to the same amount of tellurite remained viable even after 24 h (Fig. 4a). We observed a similar result when BW25113 was treated with tellurite. In the absence of ALA, colony-forming units (CFU) ml^−1^ were 1,700-fold higher than in cells grown in the presence of ALA (Fig. 4a). In both strains, ALA was equally incorporated and reached levels 400% higher than in strain BW25113 grown in M9 medium and had no effect on cell viability. A similar increase in heme content was also observed in both strains (Fig. 4a).

The increased tellurite toxicity in the presence of ALA could be caused by an accumulation of intermediates in heme biosynthesis. Indeed, we observed increased levels of proto IX, copro III and other unidentified intermediates of heme biosynthesis in BW25113 and EM2 grown with ALA and exposed to tellurite (Fig. 4b,c). In strain EM2, proto IX and copro III accumulated over time and reached a 90- and 2-fold increase after 180 min of tellurite exposure, respectively. In strain BW25113, the levels of both metabolites increased ∼3.5-fold (Fig. 4b). Because BW25113 and EM2 were treated with different tellurite concentrations, we believe that the lower accumulation of proto IX and slower cell death observed in strain BW25113 as compared to strain EM2 are due to differences in the rate of tellurite incorporation. Supporting this view, the levels of intracellular tellurite were fourfold higher in strain EM2 treated with 400 μg ml^−1^ (763.98±131.1 μg ml^−1^ of tellurium g of cell pellet^−1^) than in strain BW25113 treated with 25 μg ml^−1^ (187.04±26.5 μg ml^−1^ of tellurium g of cell pellet^−1^) at 180 min. Additionally, we acknowledge that the levels of proto IX in cells grown with ALA are two- and eightfold higher in strains BW25113 and EM2 even before tellurite exposure (Fig. 4b) than in strain BW25113 treated with tellurite alone (Fig. 3a). This indicates that under the tested conditions, proto IX *per se* is not lethal to *E. coli* and might not be solely responsible for the observed death, but that the combination of proto IX and tellurite causes cell death.

Importantly, we revealed that ALA potentiates the toxic effects of tellurite and that this occurs in both a sensitive (BW25113) and a resistant strain (EM2) to tellurite (Fig. 4a). Yet, at this point, the degree of this potentiation is unknown. To determine the minimal amount of tellurite required to inhibit growth in the presence of ALA, we challenged strains BW25113, Δ*hemN*, Δ*hemF* and EM2 with decreasing amounts of tellurite in the presence of a fixed amount of ALA (25 μg ml^−1^). In all cases, the amount of tellurite required to inhibit growth was lower when ALA was present (compare Figs 1c and 2b with Fig. 4d and Supplementary Fig. 5). To further investigate the potentiation between these two compounds, we determined the minimum concentration of ALA that inhibited cell growth when combined with tellurite. As a proof of principle, these experiments were limited to strains BW25113 and EM2. We used a fixed amount of tellurite (0.5 and 50 μg ml^−1^ for strain BW25113 and EM2, respectively) that allowed robust growth in the absence (Fig. 1b) but not in the presence of ALA (Fig. 4d), and varied the concentration of ALA. A concentration as low as 0.098 μg ml^−1^ (∼750 nM) ALA was enough to inhibit growth of strains BW25113 and EM2 (Fig. 4e and Supplementary Fig. 6). In summation, our results show that in *E. coli*, tellurite toxicity highly correlates with the accumulation of intermediates in heme biosynthesis, mainly proto IX, and that the addition of ALA further potentiates this effect.

### Tellurite exposure leads to iron deficiency

**Figure 5:**
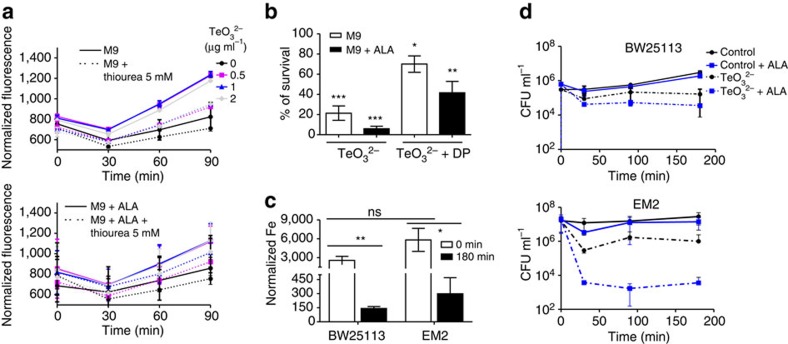
Tellurite exposure causes iron depletion and ^·^OH generation. (**a**) Strain BW25113 was grown in M9 medium supplemented or not with 25 μg ml^−1^ ALA or 5 mM thiourea, and hydroxyl radical generation was determined at the indicated time points. Tellurite concentrations that were used are indicated. Values represent the mean of three independent experiments. Error bars represent s.d. (**b**) Strain BW25113 was grown in M9 medium supplemented or not with 25 μg ml^−1^ ALA or 1 mM 2,2′-DP, and challenged with 25 μg ml^−1^ tellurite, and CFU ml^−1^ were determined after 180 min. Per cent survival is with respect to the culture at time 0 min. Values represent the mean of three biological replicates. Error bars represent s.d. *P<0.05, **P<0.001 and ***P<0.0001 as determined by *t*-test. (**c**) ICP-OES analyses. Strain BW25113 was grown in M9 medium, challenged with tellurite (25 μg ml^−1^) and intracellular iron was quantified by ICP-OES analyses after 180 min. Units for normalized Fe are μg ml^−1^ of iron g of cell pellet^−1^. Values represent the mean of three biological replicates. Error bars represent s.d. *P<0.05, **P<0.001. NS, not significant as determined by *t*-test. (**d**) Overnight cultures of strains BW25113 and EM2 were grown anaerobically and inoculated into M9 medium supplemented or not with 25 μg ml^−1^ ALA, and challenged or not with 50 or 400 μg ml^−1^ tellurite, respectively. At the indicated time points, CFU ml^−1^ were quantified. Values represent the mean of three biological replicates. Error bars represent s.d. TeO_3_^2−^: tellurite.

Previous studies showed that under aerobic conditions, tellurite can damage the iron–sulfur cluster of some enzymes^36^, suggesting that the intracellular pool of free iron could be altered because of tellurite exposure. As porphyrins are a source of several ROS^37^,^38^ and also accumulate after tellurite exposure (Fig. 3e), we speculated that iron and H_2_O_2_ could react through the Fenton reaction, generating the highly oxidizing hydroxyl radical (^·^OH), leading to cell death. Hydroxyl radical was detected in strain BW25113 challenged with tellurite and the levels increased over time. Supplementing the growth media with the ^·^OH quencher thiourea^39^ resulted in fluorescence levels similar to those observed in untreated cells (Fig. 5a). A similar result was observed in cells grown with ALA. To test directly if iron played a role in tellurite toxicity, BW25113 cells were challenged with tellurite in the presence or absence of the iron chelator 2,2′-dipyridyl (DP). After 180 min of tellurite treatment, only 20% of cells remained viable, versus 70% in media supplemented with DP. A similar trend was observed in cells grown with ALA; only 6% of cells remained viable after tellurite treatment, versus 40% when DP was present (Fig. 5b). The previous experiments showed that decreasing iron levels increased tellurite resistance. Hence, we hypothesized that *E. coli* would respond to tellurite exposure by limiting iron availability. After 180 min of tellurite exposure, the levels of intracellular iron decreased 19-fold in strain BW25113, as compared to the starter culture. A similar decrease was observed in strain EM2 (Fig. 5c). Together, our experiments show that iron is harmful for the cell when exposed to tellurite, and that *E. coli* responds by drastically decreasing iron levels. However, this defense mechanism could be detrimental for the cell. Iron is a substrate for the last step of heme biosynthesis, catalysed by ferrochetalase, and limiting iron availability after tellurite exposure could be problematic and lead to proto IX accumulation, explaining, at least in part, the observed results.

These experiments show that ^·^OH, but not H_2_O_2_ or O_2_^·−^, plays a role in tellurite toxicity. However, they do not rule out the possibility that proto IX, and perhaps other porphyrins, are also toxic. To directly test this, strains BW25113 and EM2 were grown under anaerobic conditions, where ROS are not generated, with or without 25 μg ml^−1^ ALA and challenged with 50 or 400 μg ml^−1^ tellurite, respectively. It was expected that if proto IX is toxic, then ALA should still potentiate tellurite toxicity. Strains BW25113 and EM2 remained viable when grown with or without ALA (Fig. 5d). However, as hypothesized, ALA strongly sensitized both strains to tellurite and CFU decreased 94.3% and 99.9%, respectively, as compared to the starter cultures (Fig. 5d). At the very least, this means that tellurite has an acute mechanism of toxicity that is independent of ROS and is potentiated by ALA.

## Discussion

Until now, the mechanism of tellurite resistance was unclear. To gain insights into this process, we developed an unbiased platform to identify novel mechanisms of tellurite resistance and intracellular targets. Resequencing a spontaneous *E. coli*-resistant strain predicted the heme biosynthetic pathway as a tellurite target (Supplementary Table 1 and Figs 1 and 2). Mutation of HemA at residue A31E in strain EM2 decreased ALA levels (Fig. 1d). Alignment of the amino-acid sequence of HemA from *E. coli* BW25113 and EM2 with that of other organisms showed that residue 31 is highly conserved and located at the catalytic N-terminal domain (Supplementary Fig. 7), based on the crystal structure of HemA from *Methanopyrus kandleri*^40^. Hence, replacing the small and nonpolar amino-acid alanine with a polar, charged amino acid with a bulky side chain like glutamic acid could affect the folding and stability of the domain and influence the surface involved in interactions with the substrate, affecting the production of ALA.

The other two mutations found in strain EM2 that increased tellurite resistance (Supplementary Fig. 1b) affected the genes encoding the housekeeping sigma factor RpoD and the transcription factor PspF. Mutation of RpoD residue R603 (Supplementary Table 1) has been previously isolated and selected as conferring increased fitness to ethanol stress, and is implicated in a reduction of transcriptional capacity^27^. Hence, it is predicted that the mutation of RpoD R603S has a pleiotropic effect and alters the transcriptional profile of strain EM2 as compared to strain BW25113. PspF is the transcriptional activator of the stress inducible *psp* operon^28^, which is induced in conditions of membrane stress that most likely result in the impairment of the proton motif force. We hypothesize that mutation of PspF at residue L52 alters the expression of the *psp* operon, allowing the cell to cope more promptly and efficiently to the stress generated by tellurite on the cell membrane.

Screening a barcoded *E. coli* deletion collection revealed that deleting *hemN*, required for the conversion of copro III to protoporphyrinogen IX, increased tellurite resistance (Fig. 2a.b and Supplementary Fig. 4). For many years, HemF was considered the main enzyme catalysing the conversion of copro III to protoporphyrinogen IX in aerobiosis, although no studies have addressed the individual contribution of each enzyme *in vivo*. However, recent studies suggest that HemN is the housekeeping enzyme in this process^34^, because it is functional under oxic and anoxic conditions^31^,^32^ and that under aerobic conditions the levels of HemN are fourfold higher than those of HemF^41^. This could explain why strain Δ*hemN* showed the greatest increase in tellurite resistance, as it would accumulate higher levels of copro III, limiting substrate availability for the last steps of heme biosynthesis.

**Figure 6:**
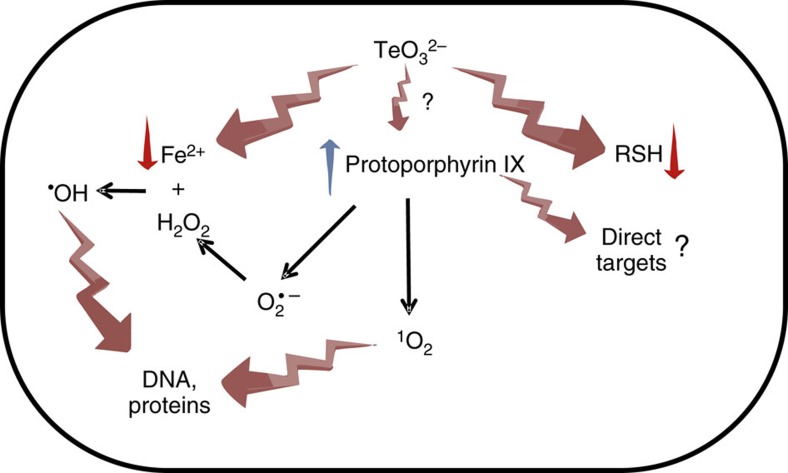
Schematic model for tellurite and proto IX toxicity in *E. coli*. Once inside the cell, tellurite depletes the pool of reduced thiols (RSHs) and targets iron–sulfur clusters. As a response, *E. coli* decreases the pool of intracellular iron, substrate for the enzyme ferrochetalase together with proto IX, causing its accumulation. Alternatively, tellurite could directly inactivate enzymes of the last two steps of heme biosynthesis (?), resulting in protoporphyrin IX accumulation. Protoporphyrin IX directly targets unidentified cellular targets (?) and also acts as a substrate for the production of superoxide (O_2_^·−^) and/or singlet oxygen (^1^O_2_) by electron or energy transfer, respectively. The O_2_^·−^ is dismutated to hydrogen peroxide (H_2_O_2_), which reacts with the free Fe^2+^ (Fenton reaction) derived from the direct action of tellurite, generating the highly toxic hydroxyl radical (^·^OH). As *E. coli* does not encode enzymes for the detoxification of ^·^OH or ^1^O_2_ and the defensive RSHs are depleted, these ROS, together with protoporphyrin IX, target intracellular macromolecules, causing cell death. TeO_3_^2−^: tellurite.

Unexpectedly, tellurite resistance arose from limiting heme biosynthesis with the trade-off of decreasing H_2_O_2_ resistance and catalase activity. Further, increased superoxide detoxification was not required (Supplementary Fig. 3a–c), indicating that neither H_2_O_2_ nor O_2_^·−^ play a key role in tellurite toxicity. This is somehow counterintuitive, as tellurite alone or combined with ALA were more efficient at killing *E. coli* under aerobic conditions (Figs 4a and 5d), suggesting that ROS is implicated, at least in part, in this process. Porphyrins can generate ROS by either electron or hydride transfer or by energy transfer, generating O_2_^·−^ and singlet oxygen (^1^O_2_)^37^,^38^, among other species. Superoxide can be further dismutated to H_2_O_2_, which can react with Fe^2+^ to generate ^·^OH ultimately. Our results show that tellurite exposure leads to ^·^OH generation and iron depletion, and that DP increases tellurite resistance (Fig. 5a–c), supporting a role for ^·^OH in tellurite toxicity. Interestingly, *E. coli* does not have dedicated enzymes able to detoxify ^·^OH or ^1^O_2_, and metabolites such as reduced thiols have been implicated in the protection against these ROS^38^. As tellurite depletes the intracellular pool of reduced thiols (Supplementary Fig. 3e), this could explain why increased levels of proto IX are toxic only in the presence of tellurite, and why ALA potentiates its toxicity (Fig. 3a,b). Our results support a model where under aerobic conditions, proto IX is a major source of ^·^OH, and most likely also of ^1^O_2_, which in the absence of any additional stress can be tolerated by the cell. However, on tellurite exposure, the intracellular levels of iron are altered and the pool of reduced thiols is depleted, fuelling the production of ^·^OH and ^1^O_2_ and depleting the defense systems against them, leading to cell death. In turn, the increased tellurite resistance observed in strains EM2, Δ*hemN* and Δ*hemF* results by limiting proto IX levels, the source of ^·^OH and ^1^O_2_ (Fig. 6).

Little is known about the toxicity of porphyrins and tellurite under anaerobic conditions; however, our results show that ALA potentiates tellurite toxicity under this condition (Fig. 5d), suggesting that porphyrins, and most likely proto IX, could also be toxic. An alternative explanation for this effect could be a direct oxidation of ALA by tellurite. Under aerobic conditions and after enolization, ALA is oxidized by molecular oxygen with the subsequent production of superoxide and hydroxyl radical^42^. It is possible that under both aerobic and anaerobic conditions, tellurite instead of oxygen could oxidize ALA, which could explain the observed cell death under anaerobic conditions (Fig. 5d). ^1^H nuclear magnetic resonance (NMR) analysis suggested that this reaction is possible, although after long periods (Supplementary Fig. 8). Whether this reaction occurs at a faster rate inside cells that could explain the rapid decrease in viability that is observed (Fig. 4a) remains to be addressed.

In *E. coli*, tellurite exposure caused the accumulation of intermediates in heme biosynthesis (Fig. 3a), mainly proto IX. Several non-exclusive mechanisms could explain this effect. Tellurite exposure caused a rapid decrease of intracellular iron (Fig. 5c), most likely as a response to prevent ^·^OH generation (Fig. 5a). However, this defense mechanism could be problematic to cope with heme biosynthesis, as iron, together with proto IX, are substrates for the last enzyme in heme biosynthesis, ferrochetalase. The result would be an accumulation of proto IX due to decreased iron availability. In turn, this could lead to further ^·^OH generation or direct damage by proto IX. In *E. coli*, H_2_O_2_ exposure also causes proto IX accumulation, and the cell responds by increasing the expression of *hemF* to ensure heme availability for the enzyme catalase^34^. However, proteomic and transcriptomic studies showed that no genes of the heme biosynthetic pathway are differentially expressed after tellurite exposure^19^,^20^, strongly suggesting that limiting iron levels causes proto IX accumulation. Further supporting the view that tellurite and H_2_O_2_ have different resistance and toxicity mechanisms in *E. coli*, DP increased tellurite resistance (Fig. 5c), while it sensitizes the cell to H_2_O_2_ (ref. 34). Another explanation for the increased levels of proto IX after tellurite exposure could be a direct damage to enzymes of the pathway. Previous studies showed that the heavy metals copper, silver, mercury and lead inhibit the activity of purified ferrochetalase and coproporphyrin III oxidase from *Rhodopseudomonas sphaeroides* and *Chromatium D*, respectively^43^,^44^, which could lead to increased levels of proto IX. Whether tellurite exerts its toxicity through a similar mechanism in *E. coli* remains to be addressed.

In recent years, the global rise of antibiotic resistance has led to an urgent need to (re)discover novel treatments to control pathogens. In the past decade, PDT using heme intermediates, including ALA, has re-emerged as an alternative as it has shown to be effective in controlling cancerous cells, viruses, fungi, parasites and both Gram-positive and -negative bacteria^24^. One major advantage for the use of ALA combined with PDT is that ALA is a prodrug and is toxic only intracellularly where it is metabolized to proto IX, which in turn acts as a source of ROS when photoactivated^24^. However, one major limitation in the use of ALA and other heme derivatives is that, in some cases, millimolar doses are required to cause an effect^45^,^46^. In this context, the results presented herein could represent a step ahead in this area, as tellurite and ALA potentiate their toxicity and only micromolar doses of both compounds are enough to kill *E. coli* (Fig. 4a,d,e). Additionally, as proto IX further accumulates in cells grown with ALA and tellurite (Fig. 4b), their combined use could improve the efficacy of PDT by providing a greater source of free radicals to kill microorganisms. Supporting this hypothesis, ALA and tellurite were more efficient at killing *E. coli* in aerobiosis (Fig. 4a), which could be explained by increased ROS generation under these conditions. It is reasonable to speculate that the combined use of ALA and tellurite could be effective in killing other organisms besides *E. coli*. Preliminary experiments showed that under the same conditions used in this study, ALA potentiated tellurite toxicity and strongly inhibited the growth of *Salmonella* Typhimurium and *S*. Typhi, and to a lesser extent of *Klebsiella pneumoniae*, however, had little or no effect over *Pseudomonas aeruginosa* and a clinical strain of *E*. *coli* (Supplementary Fig. 9). This was somehow expectable, as higher concentrations of ALA and/or tellurite might be required to achieve a similar effect in different organisms, which must be determined experimentally. Furthermore, designing efficient delivery methods of ALA/tellurite adducts or synthesizing alloys between these two compounds could further improve this process and expand its use to other fields.

## Methods

### Bacterial strains and growth conditions

All strains are derivatives of *E. coli* K-12 strain BW25113 (National Institute of Genetics, Microbial Genetics Laboratory, NBRP, Japan). Strains EM40, EM41 and EM2 were isolated after 3, 13 or 26 aerobic serial passages (one serial passage=24 h) of strain BW25113 in LB medium with increasing tellurite concentrations up to 400 μg ml^−1^ (Fig. 1a). Strains were isolated by single-colony purification. The genomes of strains BW25113, EM41, EM42 and EM2, as well as the identification of mutations in each strain were determined by Macrogen Inc. (South Korea) using the Illumina HiSeq 2000 platform. Single-nucleotide polymorphisms in comparison with the reference genome of *E. coli* strain BW25113 (GenBank: CP009273.1) are deposited at doi: 10.6084/m9.figshare.4747402 (Supplementary Data 2). Strains Δ*hemN* and Δ*hemF* are derivatives of the Keio collection^33^. Strain EM42 was generated by replacing the wild-type allele with *hemA*[31E] in strain BW25113 (EM2 DNA was used as a template for PCR reactions). Strains EM43, EM44, EM 64, EM70 and EM71 were generated by replacing the mutant gene with the corresponding wild-type allele in strain EM2 (BW25113 DNA was used as a template for PCR reactions). To achieve this, the different genes were amplified using the primers listed in Supplementary Table 2 and denoted as GIBSON_ followed by the name of the gene to be amplified. The resultant PCR products were cloned into plasmid pKD3 (ref. 47) using Gibson assembly^48^. Plasmid pKD3 was amplified using primers Gibson_pKD3F and Gibson_pKD3R. The resulting PCR product was used in all reactions as the vector backbone. The resulting plasmids were used as templates to amplify the gene of interest and the selection marker (*caf*) using the primers listed in Supplementary Table 2 (named ‘wanner_' followed by the name of the gene to be amplified). All strains were constructed using λ red recombination^47^ of the corresponding PCR products into strains EM2 or BW25113. Incorporation of the desired mutation in the chromosome was confirmed by PCR and DNA sequencing.

Growth curves were conducted in M9 medium^49^ plus 0.2% glucose containing or not potassium tellurite (Sigma-Aldrich), H_2_O_2_ (Merck) supplemented or not with 25 μg ml^−1^ ALA (Sigma-Aldrich), as indicated in each figure, in 48-well plates (Nunc). Growth was started by adding 10 μl of an overnight culture of each strain grown in M9 medium into a well containing 990 μl (1:100 dilution) of the medium indicated in each figure. Growth was conducted at 37 °C in a Tecan INFINITE M200 Pro with constant shaking at 141.9 r.p.m. and the optical density (OD) at 600 nm was monitored every 15 min for 24 h. Since tellurite is reduced to tellurium inside cells (Supplementary Fig. 10), OD 600 nm might be overestimated in some cases, impeding the accurate calculation of specific growth rates and half-maximal inhibitory concentration values.

For cell viability experiments, overnight cultures grown in M9 medium were diluted (1:100) into 250 ml flasks containing 25 ml of M9 medium with or without tellurite, H_2_O_2_, 1 mM 2,2′-DP (Sigma-Aldrich), supplemented or not with 25 μg ml^−1^ ALA (as indicated in each figure) and CFU were determined at the indicated time points. Cultures were grown at 37 °C with an agitation of 250 r.p.m. For anaerobic experiments (Fig. 5d), cells were grown inside an anaerobic chamber (Coy Laboratory Products) at 37 °C with an agitation of 125 r.p.m. Growth was started as described above but under anaerobic conditions. All solutions were equilibrated inside the anaerobic chamber for at least 1 week before conducting the experiments.

### Chemical genomic profiling of tellurite with *E. coli*

Pooled competition experiments of the barcoded *E. coli* deletion collection^30^ were grown in the presence of potassium tellurite (0.63 μg ml^−1^) or a DMSO (solvent). Each pooled competition was grown in 200 μl cultures (*n*=3) at 37 °C for 24 h in a TECAN M1000. After 24 h, genomic DNA was extracted using the Invitrogen PureLink 96-well genomic DNA Extraction Kit (Cat No. K1821-04 A). Strain specific barcodes from each culture were amplified using indexed primers designed for multiplexed Illumina sequencing. The forward primer contained the Illumina specific P5 sequence, a 10 bp index tag (x's), and the 19 bp *E. coli* deletion collection common priming site: 5′-AATGATACGGCGACCACCGAGATCTACACTCTTTCCCTACACGACGCTCTTCCGATCTxxxxxxxxxxAATCTTCGGTAGTCCAGCG-3′. The reverse primer contained the Illumina-specific P7 sequence and 20 bp *E. coli* common priming site: 5′-CAAGCAGAAGACGGCATACGAGCTCTTCCGATCTTGTAGGCTGGAGCTGCTTCG-3′. Barcodes were amplified by PCR, pooled, gel purified and quantified by quantitative PCR as described^50^. For barcode sequencing, samples were run on an Illumina HiSeq2500 in rapid run mode for 50 cycles at a loading concentration of 15 pM. The resulting Fastq file was used for analysis of sensitive and resistant mutants. We compared normalized counts to a solvent (DMSO) control to identify compound specific responses among gene deletion mutants (chemical genetic interaction score). Sequence data was processed using BEANcounter (https://github.com/csbio/BEAN-counter) and EdgeR^51^.

### Extraction and measurement of intracellular ALA and heme

Overnight cultures of the indicated strains grown in M9 medium were diluted (1:100) into 500 ml flasks containing 150 ml M9 medium supplemented or not with 25 μg ml^−1^ ALA. Cultures were grown at 37 °C to an OD 600 nm of 0.4 and intracellular ALA was extracted from a 100 ml aliquot as described previously^52^ and quantified using Ehrlich's reagent (Sigma-Aldrich) by following absorbance at 553 nm. Intracellular heme was extracted from 1 ml of the same cultures as described previously^53^ and fluorescence was determined in a Tecan INFINITE M200 Pro with an excitation wavelength of 400 nm and emission wavelengths of 610 and 660 nm. For ALA and heme, values were normalized by the OD 600 nm of the culture.

### Porphyrin extraction and quantification

Overnight cultures of the indicated strains grown in M9 medium were diluted (1:100) into 2 l of M9 medium supplemented or not with 25 μg ml^−1^ ALA and grown aerobically at 37 °C to an OD 600 nm of 0.4. At this point, 25 μg ml^−1^ tellurite was added to the cultures and porphyrins were extracted from 1 l (cells grown in M9 medium) or 400 ml (cells grown in M9 medium with ALA) of the cultures at the indicated time points as described previously^54^.

Porphyrins were analysed and quantified as described by Tatsumi and Wachi^45^ using a 1,200 series high-performance liquid chromatography (HPLC) system (Agilent Technologies) including a degasser (G1322A), a quaternary pump (G1311A), a variable wavelength detector (G1314B) and a manual sampler. The injection volume was 20 μl. The LC separation was performed on an Agilent Zorbax Eclipse XDB-C18 column (4.6 × 150 mm^2^, 5 μm) eluted for 45 min at a flow rate of 1 ml min^−1^ with a linear gradient from 50% solvent A (10% acetonitrile in 1 M ammonium acetate; pH 5.1), 50% solvent B (methanol–acetic acid 10:1, v v^−1^) to 100% solvent B, followed by elution with 100% solvent B for 5 min. The porphyrin composition of each sample was analysed following the absorbance at 300 nm. Commercially available standards of coproporphyrin III and proto IX (Frontier Scientific) were used to identify and quantify each compound from the different samples. Values were normalized by mg of protein as determined by the Bradford method^55^.

### Biochemical determinations

For all biochemical determinations, overnight cultures of the indicated strains grown in M9 medium were inoculated into 250 ml flasks containing 125 ml of M9 medium supplemented or not with 25 μg ml^−1^ ALA (indicated in each figure). Cultures were grown aerobically at 37 °C with an agitation of 250 r.p.m. to an OD 600 nm of 0.4. At this time, cells were treated with either tellurite or H_2_O_2_, as indicated in each figure. At each of the indicated time points, a 25 ml aliquot was centrifuged at 8,000 *g* at 4 °C for 5 min and the cell pellet was washed two times with 5 ml of 50 mM Tris-HCl (pH 7.4). The cell pellet was suspended in 500 μl of 50 mM Tris-HCl (pH 7.4) and cells were disrupted by sonication. For catalase activity, cell pellet was suspended in 50 mM phosphate saline buffer (pH 7.4). Cell-free extracts recovered after centrifugation at 14,000 *g* at 4 °C for 20 min were used to determine the different activities. For all cases, OD was monitored using a Tecan INFINITE M200 Pro. Values were normalized by mg of protein as determined by the Bradford method^55^.

### Catalase activity

Catalase activity was determined in 200 μl reactions using 10 μl of cell-free extract as described previously^56^ by following H_2_O_2_ consumption at 240 nm. Catalase activity was defined as nmoles H_2_O_2_ consumed min^−1^ mg protein^−1^.

### Superoxide dismutase activity

Activity was determined in 200 μl reactions using 10 μl of cell-free extract in buffer 50 mM Tris-HCl (pH 7.4), 1 mM EDTA with 25 μM nitroblue tetrazolium (Sigma-Aldrich), 50 μM xanthine and 0.25 μg μl^−1^ xanthine oxidase (Sigma-Aldrich). Superoxide dismutase activity activity was monitored following NTB oxidation at 412 nm as described previously^56^. One unit (U) of superoxide dismutase activity activity was defined as the amount of enzyme required to inhibit in 50% NBT oxidation min^−1^ mg protein^−1^.

### TR activity

TR activity was determined in 200 μl reactions using 10 μl of cell free extract in buffer 50 mM Tris-HCl (pH 7.4), 1 mM tellurite, 1 mM NAD(P)H (Sigma-Aldrich) and 1 mM β-mercaptoethanol (Sigma-Aldrich). TR activity was monitored following the appearance of tellurium at 500 nm as described previously^57^. One unit (U) of TR activity was defined as the amount of enzyme required to increase the OD 500 nm in 0.001 min^−1^ mg protein^−1^.

### Total thiol content

Total thiol content was quantified using Ellman's reagent 5,5′-dithiobis-(2-nitrobenzoic acid) (Sigma-Aldrich) as described previously^58^ by following TNB^−2^ generation at OD 412 nm. Values were normalized by mg protein.

### Inductively coupled plasma-optical emission spectrometry

Inductively coupled plasma-optical emission spectrometry (ICP-OES) analysis was performed using a Perkin-Elmer optima 2000 DV apparatus in the axial viewing mode. The wavelength corresponding to tellurium was 214,281 nm. Calibration curves for tellurium and iron (in 2% ultrapure HNO_3_) were constructed. Briefly, overnight cultures of the indicated strains grown in M9 medium were inoculated (1:100 dilution) into 250 ml flasks containing 125 ml of M9 medium. Cultures were grown aerobically at 37 °C with an agitation of 250 r.p.m. to an OD 600 nm of 0.4. At this time, cells were treated with tellurite. At the indicated time points, a 25 ml aliquot was centrifuged at 8,000 *g* at 4 °C for 5 min and the cell pellet was washed twice with 5 ml of 50 mM Tris-HCl (pH 7.4). The cell pellet was weighed, and suspended in 8 ml of 2% HNO_3_ and subjected to ICP-OES analyses. Values were normalized by the weight of the cell pellet.

### ^1^H NMR analysis

The analysis was performed on a Bruker Avance 400 MHz spectrometer (400.133 MHz for ^1^H) equipped with a 5-mm multinuclear broad-band dual probe head incorporating a z-gradient coil as described previously^59^. All the measurements were carried out in M9 medium with 5 mg ALA and with or without 5 mg tellurite. Chemical shifts were calibrated with respect to the solvent signal (δ=7.26 p.p.m. for proton residual solvent) and referenced to TMS.

### Hydroxyl radical generation

To determine hydroxyl radical generation, overnight cultures of strain BW25113 grown in M9 medium were inoculated (1:100 dilution) into 125 ml flasks containing 25 ml of M9 medium supplemented or not with 25 μg ml^−1^ ALA (indicated in each figure). Cultures were grown aerobically at 37 °C with an agitation of 250 r.p.m. to an OD 600 nm of 0.4. At this time, the probe hydroxyphenyl fluorescein was added to the culture (5 μM final concentration) and cells were grown for 20 min. Cells were washed twice with 25 ml of 50 mM Tris-HCl pH 7.4 to remove excess probe. Following, the cell pellet was suspended in 25 ml of M9 medium, and 190 μl aliquots were added to individual wells of a 96 well plate. At this point, the growth medium was supplemented with the indicated concentrations of tellurite, 25 μg ml^−1^ ALA or 5 mM thiourea (Merck), and growth was continued in a Tecan INFINITE M200 Pro at 37 °C. Hydroxyl radical production was followed by measuring the fluorescence of the probe hydroxyphenyl fluorescein at the indicated time points with an excitation wavelength of 490 nm and an emission wavelength of 525 nm. Values were normalized by OD 600 nm.

### Data availability

The chemical genomic profiling and single-nucleotide polymorphism data sets (Supplementary Data 1 and 2, respectively) generated during and/or analysed in the current study have been deposited in Figshare with the identifier doi: 10.6084/m9.figshare.4747402 (ref. 60). The authors declare that all other relevant data supporting the findings of this study are available within the paper and its Supplementary Information files, or from the corresponding authors on request.

## Additional information

**How to cite this article:** Morales, E. H. *et al*. Accumulation of heme biosynthetic intermediates contributes to the antibacterial action of the metalloid tellurite. *Nat. Commun.* **8,** 15230 doi: 10.1038/ncomms15230 (2017).

**Publisher's note:** Springer Nature remains neutral with regard to jurisdictional claims in published maps and institutional affiliations.

## Supplementary Material

Supplementary Information Supplementary Figures and Supplementary Tables

Supplementary Data 1 Chemical genomics of tellurite. Positive or negative chemical genetic interaction scores represent increased or decreased tellurite resistance, respectively.

Supplementary Data 2 Single nucleotide polymorphisms found in evolved strains EM40, EM41, and EM2.

## Figures and Tables

**Table 1:** *E. coli* strains used in this study.

**Strain**	**Relevant genotype**	**Source**
BW25113	*lacI*q *rrnB*T14 Δ*lacZ*WJ16 *hsdR*514 Δ*araBAD*AH33, Δ*rhaBAD*LD78	Keio collection^31^
EM2	BW25113 *yigE*[A22V] *rpoD*[R603S] *pspF*[L52H] *hemA*[A31E] *hemL*[A118V]	This study
Δ*hemN*	BW25113 Δ*hemN*::*aph*	Keio collection^31^
*hemN*^+^	Δ*hemN* complemented in *cis*	This study
Δ*hemF*	BW25113 Δ*hemF*::*aph*	Keio collection^31^
EM40	BW25113 *yigE*[A22V]	This study
EM41	BW25113 *yigE*[A22V] *rpoD*[R603S]	This study
EM42	BW25113 *hemA*[A31E]	This study
EM43	EM2 *hemA* wild type	This study
EM44	EM2 *hemL* wild type	This study
EM53	BW25113 *yigE*[A22V] *rpoD*[R603S]	This study
EM64	EM2 *rpoD* wild type	This study
EM70	EM2 *pspF* wild type	This study
EM71	EM2 *rpoD pspF* wild type	This study
