# Supplementary material for: Accumulation of heme biosynthetic intermediates contributes to the antibacterial action of the metalloid tellurite
Source: Nat Commun. 2017 May 11;8:15320. doi: 10.1038/ncomms15320 (PMC5437285; doi:10.1038/ncomms15320)
Supplement: Supplementary Information — Supplementary Figures and Supplementary Tables [file ncomms15320-s1.pdf]

Supplementary Figure 1.

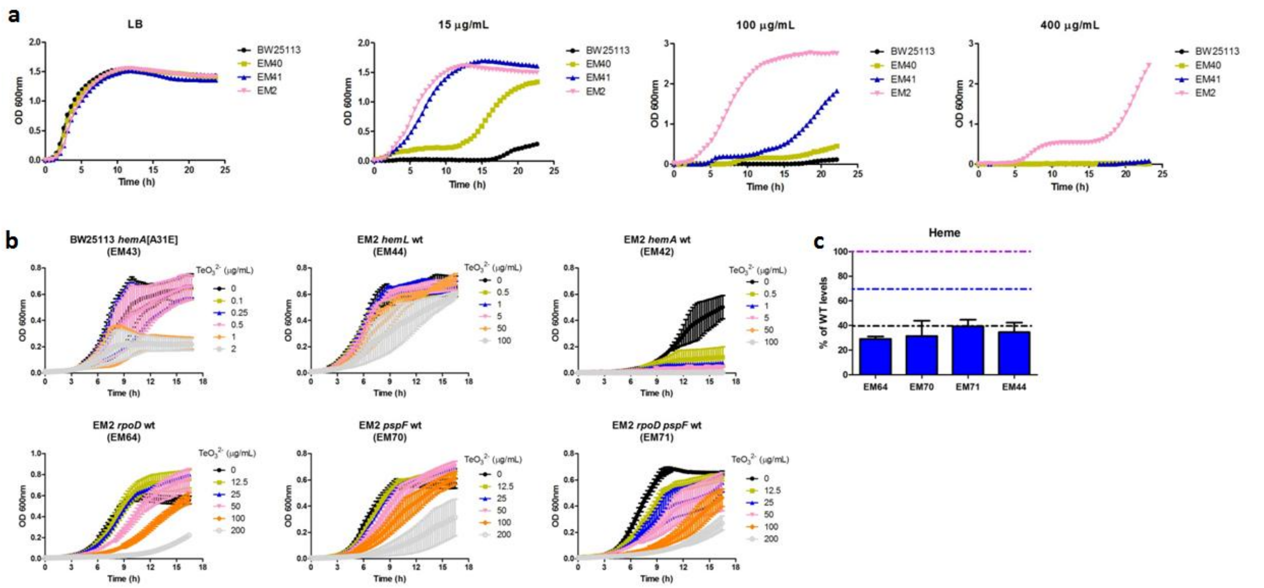

Selection of mutants conferring increased tellurite resistance. a) Representative growth curves of strains BW25113, EM40, EM41 and EM2 in LB medium in the presence of the indicated tellurite concentrations. **b**) Representative growth curves of strains EM42, EM43, 44, 64, 70, and 71 in M9 medium in the presence of the indicated tellurite concentrations. Values represent the mean of 3 biological replicates. In all figures, error bars represent SD. **c**) Strains EM 44, EM64, EM70 and EM71 were grown in M9 medium and the heme content was measured in cell extracts. For comparison, the level of heme in strains BW25113 (magenta line), EM43 (blue line) and EM2 (black line) are shown. Values represent the mean of three biological replicates. Error bars represent SD. TeO_3^{2-} : tellurite.

Supplementary Figure 2.

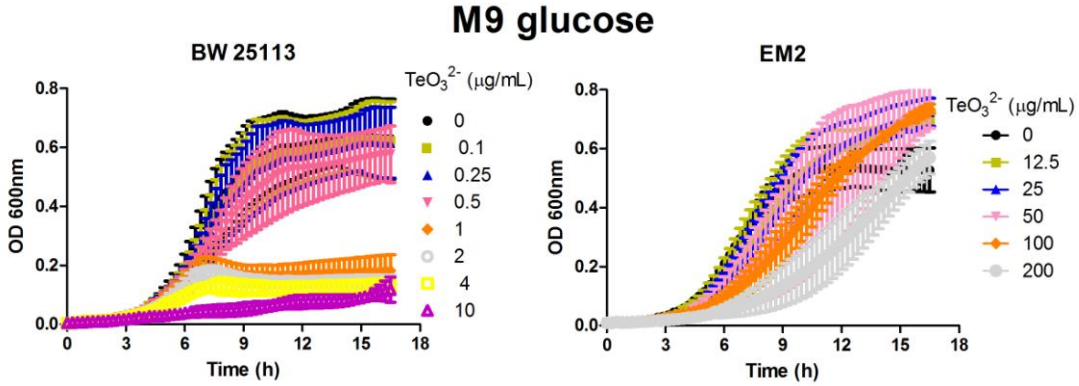

Representative growth curves of strains BW25113 and EM2 with tellurite. Representative growth curves of strains BW25113 and EM2 in M9 medium in the presence of the indicated tellurite concentrations. Values represent the mean of 3 biological replicates. Error bars represent SD. TeO_3^{2-} : tellurite.

Supplementary Figure 3.

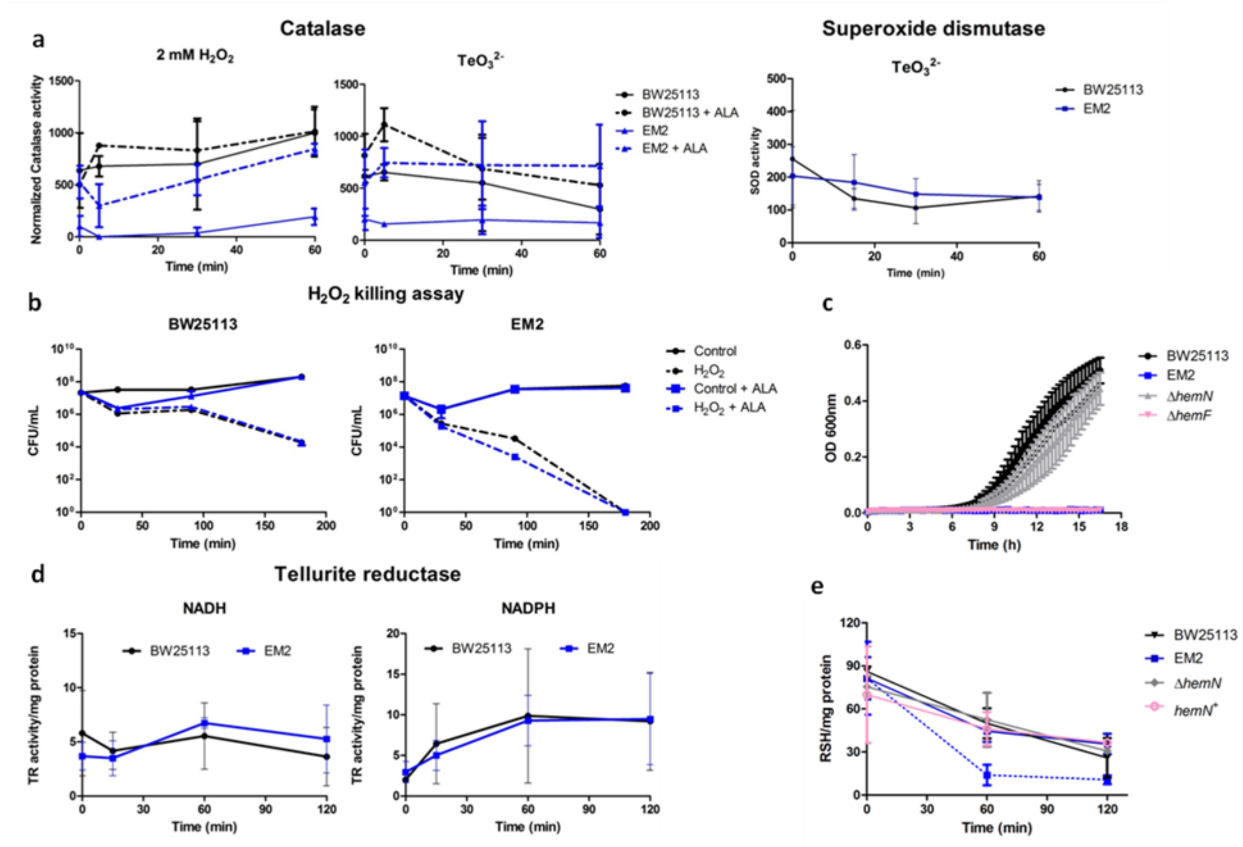

Increased thiols, H_2O_2 detoxification or resistance are not required for tellurite resistance. a) Strains BW25113 and EM2 were grown in M9 medium with or without 25 µg/mL ALA to an OD 600nm of ˜0.4 and treated with 2 mM H_2O_2 (left panel) or tellurite (middle and right panel, 25 µg/mL for strain BW25113 and 400 µg/mL for strain EM2) and catalase (left and middle panels) or superoxide dismutase (SOD, right panel) activity were determined at the indicated time points. SOD activity was normalized by mg of protein. Values represent the mean of three biological replicates. Error bars represent SD. **b**) Overnight cultures of strains BW25113 and EM2 were inoculated into M9 medium supplemented or not with 25 µg/mL ALA, challenged or not with 2 mM H_2O_2 and CFU/mL were quantified at the indicated time points. Values represent the mean of three biological replicates. Error bars represent SD. **c**) Representative growth curves of strains BW25113, EM2, Δ*hemN* and Δ*hemF* in M9 medium with 0.3 mM H_2O_2. Values represent the mean of three biological replicates. Error bars represent SD. **d**) Strains BW25113 and EM2 were grown as in **a**), exposed to tellurite and tellurite reductase (TR) activity was determined with NADH or NADPH as cofactor. Values represent the mean of three biological replicates. Error bars represent SD. **e**) Strains BW25113, Δ*hemN*, *hemN*$^+$ and EM2 were grown as in **a**), exposed to 25 µg/mL tellurite and total intracellular thiols were determined and normalized by mg of protein. Strain EM2 was also treated with 400 µg/mL tellurite, and thiols were determined (blue dashed line). Values represent the mean of three biological replicates. Error bars represent SD.TeO_3^{2-} : tellurite; RSH: µM thiols.

Supplementary Figure 4.

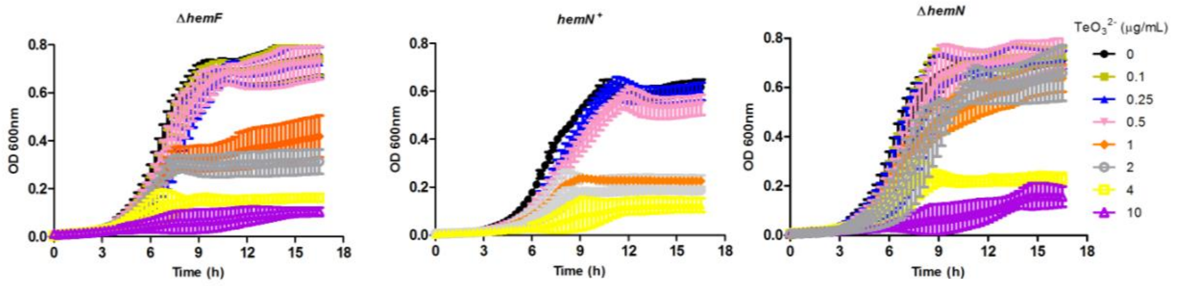

Representative growth curves of strains Δ*hemF, hemN*[+] and Δ*hemN* with tellurite. Representative growth curves of strains Δ*hemF, hemN*[+] and Δ*hemN* in M9 medium in the presence of the indicated tellurite concentrations. Values represent the mean of 3 biological replicates. Error bars represent SD. TeO_3^{2-} : tellurite.

Supplementary Figure 5.

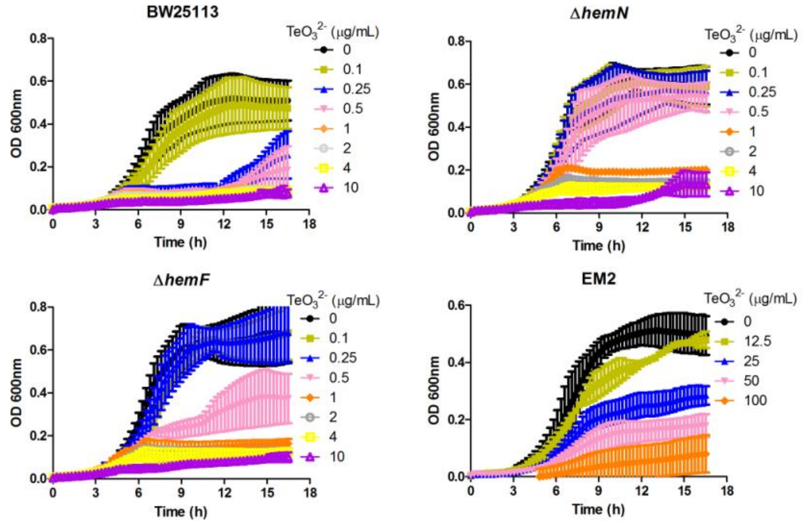

Representative growth curves of strains BW25113, EM2, Δ*hemN* and Δ*hemF* in M9 medium supplemented with 25 µg/mL ALA and varying tellurite concentrations. Values represent the mean of 3 biological replicates. TeO_3^{2-} : tellurite.

Supplementary Figure 6.

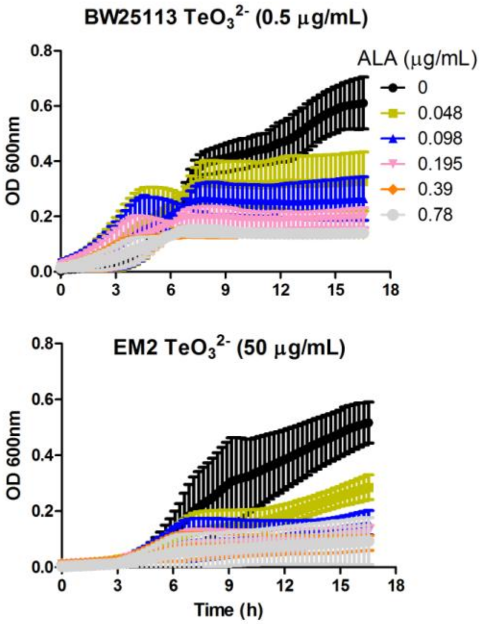

Representative growth curves of strains BW25113 and EM2 in M9 medium supplemented with the indicated concentrations of tellurite and varying concentrations of ALA. Values represent the mean of 3 biological replicates. TeO_3^{2-} : tellurite.

Supplementary Figure 7.

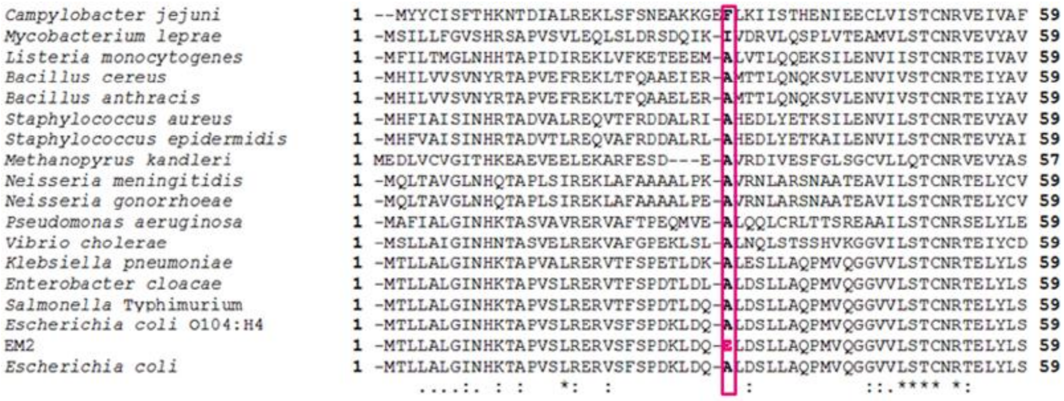

Conservation of HemA residue mutated in strain EM2. The amino acid sequences of HemA from strain EM2 was aligned against that of other organisms. Residue that was mutated in strain EM2 is indicated by a magenta box along all the sequences.

Supplementary Figure 8.

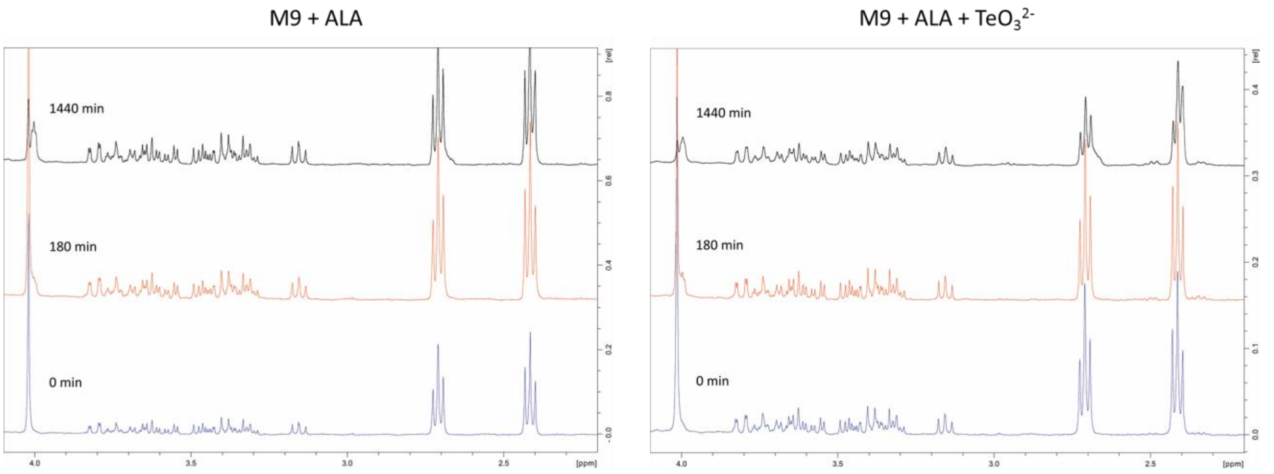

^{1}H NMR spectra of ALA in M9 medium with or without tellurite. All the measurements were carried out in M9 medium with 5 mg ALA and with or without 5 mg tellurite. TeO_3^{2-} : tellurite.

Supplementary Figure 9.

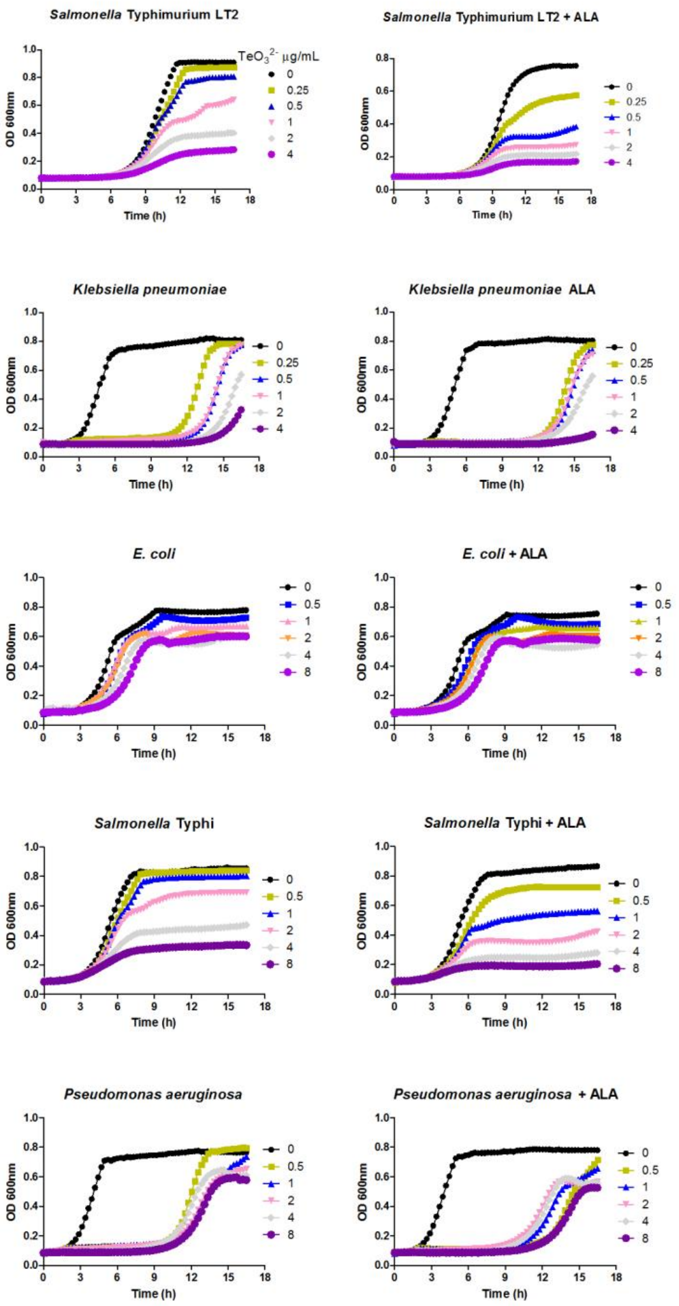

Evaluation of the effect of ALA and tellurite on selected pathogens. Representative growth curves of selected pathogens in M9 medium supplemented with 25 µg/mL ALA in the presence of the indicated tellurite concentrations. TeO_3^{2-} : tellurite.

Supplementary Figure 10.

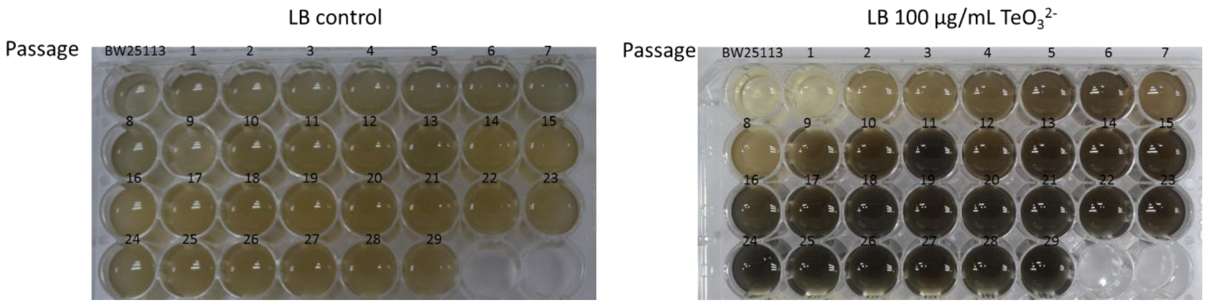

***E. coli* growth in the presence of high tellurite concentrations results in black deposits of elemental tellurium.** Representative growth curves in LB medium containing or not 100 µg/mL tellurite of colonies isolated from each passage during directed evolution experiment. Number at the top of each well indicates the passage at which the corresponding colony was isolated. Strains EM40, EM41 and EM2 were isolated at passages 3, 13, and 26, respectively. Blackening of the growth culture is due to tellurite reduction to tellurium. TeO_3^{2-} : tellurite.

Supplementary Table 1. Nonsynonymous mutations found in evolved strains.

Strain(s)	Gene	Nucleotide change	Amino acid change
EM 40, EM41, EM2	*yigE*	GCC → GTC	A22V
EM 41, EM2	*rpoD*	CGT → AGT	R603S
EM 2	*pspF*	CTC → CAC	L52H
EM 2	*hemA*	GCG → GAG	A31E
EM 2	*hemL*	GCG → GTG	A118V

Supplementary Table 2. Primers used in this study.

Primer name	SEQUENCE 5' → 3'	Amplified product
Gibson_pKD34F	GTGTAGGCTGGAGCTGCT	Plasmid pKD3
Gibson_pKD34R	AATCGCTCAAGACGTGTAATG	
Gibson_hemAF	CACGTCTTGAGCGATTATGACCCTTTTAGCACTCGG	*hemA* for cloning in plasmid pKD3
Gibson_hemAR	AGCTCCAGCCTACACTACTCCAGCCCGAGGCTG	
Gibson_hemLF	CACGTCTTGAGCGATTATGAGTAAGTCTGAAAATCTTTAC	*hemL* for cloning in plasmid pKD3
Gibson_hemLR	AGCTCCAGCCTACACTCACAACTTCGCAAACAC	
Gibson_rpoDF	ATTACACGTCTTGAGCGATTCACCAACCTCATGAAATAAG	*rpoD* for cloning in plasmid pKD3
Gibson_rpoDR	GAAGCAGCTCCAGCCTACACTTAATCGTCCAGGAAGCTAC	
Gibson_pspFF	ATTACACGTCTTGAGCGATTTTAGCGAATTACACTAACAAG	*pspF* for cloning in plasmid pKD3
Gibson_pspFR	GAAGCAGCTCCAGCCTACACCTAAATCTGGTGCTTTTTC	
Gibson_hemNF	ATTACACGTCTTGAGCGATTCGTAGCCGCCAGAGACGC	*hemN* for cloning in plasmid pKD3
Gibson_hemNR	GAAGCAGCTCCAGCCTACACTTAAATCACCCGAGAGAACTGCTGC	
wanner_muthemAF	GATGCAAGCAGACTAACCCT	*hemA*::cat cassette for restoring *hemA*
wanner_hemAR	AAATGCACCCTGTAAAAAAAGAAAATGATGTACTGC ATATGAATATCCTCCTTAG	
wanner_muthemLF	GCACCAGTACAAGCAGCCTG	*hemL*::cat cassette for restoring *hemL*
wanner_hemLR	AGGCGTTCACGCCGCATCCGACAAACCATGCTGGACATATGAATAT CCTCCTTAG	
wanner_hemN2F	CGTAGCCGCCAGAGACGC	*hemN*::cat cassette for restoring *hemN*
wanner_hemNR	GTTTTCTACTTTGTAAACGAAGCGCCATTCACTATCATATGAATATCC TCCTTAG	
wanner_rpoD2F	CACCAACCTCATGAAATAAG	*rpoD*::cat cassette for restoring *rpoD*
wanner_rpoDR	CCGGGTGCGGCGTAACGCCTGATCCGGCCTACCGACATATGAATATC CTCCTTAG	
wanner_pspF2F	TTAGCGAATTACACTAACAAG	*pspF*::cat cassette for restoring *pspF*
wanner_pspFR	ACGCCGCATCCGGCAAGTTGTATTGCTCAACTTCGCATATGAATATCCT CCTTAG	
